# Aggregate Index of Systemic Inflammation (AISI), Disease Severity, and Mortality in COVID-19: A Systematic Review and Meta-Analysis

**DOI:** 10.3390/jcm12144584

**Published:** 2023-07-10

**Authors:** Angelo Zinellu, Panagiotis Paliogiannis, Arduino A. Mangoni

**Affiliations:** 1 Department of Biomedical Sciences, University of Sassari, 07100 Sassari, Italy; azinellu@uniss.it; 2 Anatomical Pathology and Histology, University Hospital (AOU) of Sassari, 07100 Sassari, Italy; ppaliogiannis@uniss.it; 3 Department of Medicine, Surgery and Pharmacy, University of Sassari, 07100 Sassari, Italy; 4 Discipline of Clinical Pharmacology, College of Medicine and Public Health, Flinders University, Bedford Park, SA 5042, Australia; 5 Department of Clinical Pharmacology, Flinders Medical Centre, Southern Adelaide Local Health Network, Bedford Park, SA 5042, Australia

**Keywords:** aggregate index of systemic inflammation, AISI, COVID-19, disease severity, mortality, biomarkers, inflammation

## Abstract

Combined indices of different haematological cell types appear to be particularly promising for investigating the link between systemic inflammation and coronavirus disease 2019 (COVID-19). We conducted a systematic review and meta-analysis to assess the aggregate index of systemic inflammation (AISI), an emerging index derived from neutrophil, monocyte, platelet, and lymphocyte counts, in hospitalized COVID-19 patients with different disease severity and survival status. We searched electronic databases between the 1st of December 2019 and the 10th of June 2023 and assessed the risk of bias and the certainty of evidence. In 13 studies, severe disease/death was associated with significantly higher AISI values on admission vs. non-severe disease/survival (standard mean difference (SMD) = 0.68, 95% CI 0.38 to 0.97, *p* < 0.001). The AISI was also significantly associated with severe disease/death in five studies reporting odds ratios (4.39, 95% CI 2.12 to 9.06, *p* ˂ 0.001), but not in three studies reporting hazard ratios (HR = 1.000, 95% CI 0.999 to 1.002, *p* = 0.39). The pooled sensitivity, specificity, and area under the curve values for severe disease/death were 0.66 (95% CI 0.58 to 0.73), 0.78 (95% CI 0.73 to 0.83), and 0.79 (95% CI 0.76 to 0.83), respectively. Our study has shown that the AISI on admission can effectively discriminate between patients with different disease severity and survival outcome (PROSPERO registration number: CRD42023438025).

## 1. Introduction

There is very good evidence that immune dysregulation and a state of excessive systemic inflammation predispose patients with coronavirus disease 2019 (COVID-19) to homeostatic alterations in various organs and tissues and an increased risk of severe clinical manifestations, prolonged hospitalization, and in-hospital mortality [1,2,3,4,5,6,7,8,9,10]. Although these observations have been instrumental in the identification of safe and effective immunomodulatory and anti-inflammatory treatment strategies [11,12], they have also stimulated the search for robust biomarkers of excessive inflammatory response for early risk stratification and appropriate management in this patient group [13,14]. Concomitantly, abnormalities in specific blood cell types, particularly, neutrophilia, lymphopenia, and thrombocytopenia, have led to studies reporting significant associations between derived indices, particularly the neutrophil-to-lymphocyte ratio (NLR), and severe disease and mortality [15,16,17,18]. The potential clinical use of another haematological index, the aggregate index of systemic inflammation (AISI, calculated by multiplying the neutrophil, monocyte, and platelet count, and dividing the product by the lymphocyte count), originally developed by Paliogiannis et al. in surgical patients [19], has been increasingly investigated in other disease states as well as COVID-19 [20,21,22,23,24,25].

Given the significant temporal changes in viral variants, pharmacological treatments, and vaccines during the COVID-19 pandemic, we critically appraised, by means of systematic review and meta-analysis, the potential utility of the AISI in discriminating between hospitalized COVID-19 patients with different severity and survival outcomes. We hypothesised that severe disease or in-hospital mortality were associated with higher AISI values at the time of admission. We also investigated possible associations between the effect size of the AISI values and various study and patient characteristics.

## 2. Materials and Methods

We searched Scopus, Web of Science, and PubMed for articles published from the 1st of December 2019 to the 10th of June 2023 using the following terms: (1) “AISI”, (2) “aggregate index of systemic inflammation”, (3) “COVID-19”, (4) “2019-nCoV”, (5) “SARS-CoV-2”, and (6) “coronavirus disease 2019”. We also hand searched the reference lists of each article for additional studies. The inclusion criteria were as follows: (a) investigating hospitalized patients with COVID-19 with different severity and survival outcomes; (b) reporting continuous data on AISI values in COVID-19 patients; (c) reporting multivariate adjusted odds ratio (OR) or hazard ratio (HR) with 95% confidence intervals (CI) for disease severity and/or mortality; (d) reporting prognostic accuracy (area under the receiver operating characteristic curve, AUROC, with 95% CIs); (e) full-text available; (f) age of participants ≥18 years; and (g) English language used. Two investigators independently reviewed the abstracts and full-text articles, with a third involved in case of disagreement.

The following information was extracted from each article: participant age and sex, publication year, sample size, study design, study country and continent, clinical endpoint studied (measures of disease severity and/or survival status), AISI, AUROC, sensitivity, specificity, cut-off, true positive (TP), false positive (FP), false negative (FN), and true negative (TN) values.

The Joanna Briggs Institute Critical Appraisal Checklist for case-control studies was used to assess the risk of bias (studies addressing <50%, ≥50% and <75%, and ≥75% of checklist items were adjudicated as having a high, moderate, and low risk, respectively [26]). The certainty of evidence was evaluated with the Grades of Recommendation, Assessment, Development and Evaluation (GRADE) Working Group system [27]. We complied with the PRISMA 2020 statement (Supplementary Tables S1 and S2) [28], and registered our study in the International Prospective Register of Systematic Reviews (PROSPERO registration number: CRD42023438025).

### Statistical Analysis

Forest plots of continuous AISI values were generated using standardized mean differences (SMDs) and 95% CIs to assess differences between patients with non-severe disease/survivors (NSDS) and those with severe disease/non-survivors (SDNS) (*p* < 0.05 for statistical significance). Additional forest plots were generated using the ORs or HRs and 95% CIs of the multivariate associations between the AISI and disease severity and survival status. Heterogeneity was assessed using the Q statistic (*p* < 0.10 for statistical significance) and random-effect models were used accordingly [29]. Sensitivity analyses assessed the stability of the effect size [30]. Publication bias was assessed using (1) Begg’s adjusted rank correlation test, (2) Egger’s regression asymmetry test (*p* < 0.05 for statistical significance) [31,32], and (3) the “trim-and-fill” method [33]. Univariate meta-regression and subgroup analyses investigated associations between the effect size and age, sex, number of study participants, study continent, publication year, and study endpoint.

We generated a summary receiver operating characteristic (SROC) curve [34], used empirical Bayes estimates, and calculated the pooled sensitivity and specificity. The HSROC model was also used to account for heterogeneity [34,35,36,37]. Publication bias was assessed using the Deeks method [38]. The relationship between prior probability, likelihood ratio, and posterior test probability was assessed using the Fagan’s nomogram plot [39]. Analyses were performed using Stata 14 (StataCorp LLC, College Station, TX, USA).

## 3. Results

### 3.1. Study Selection

**Figure 1:**
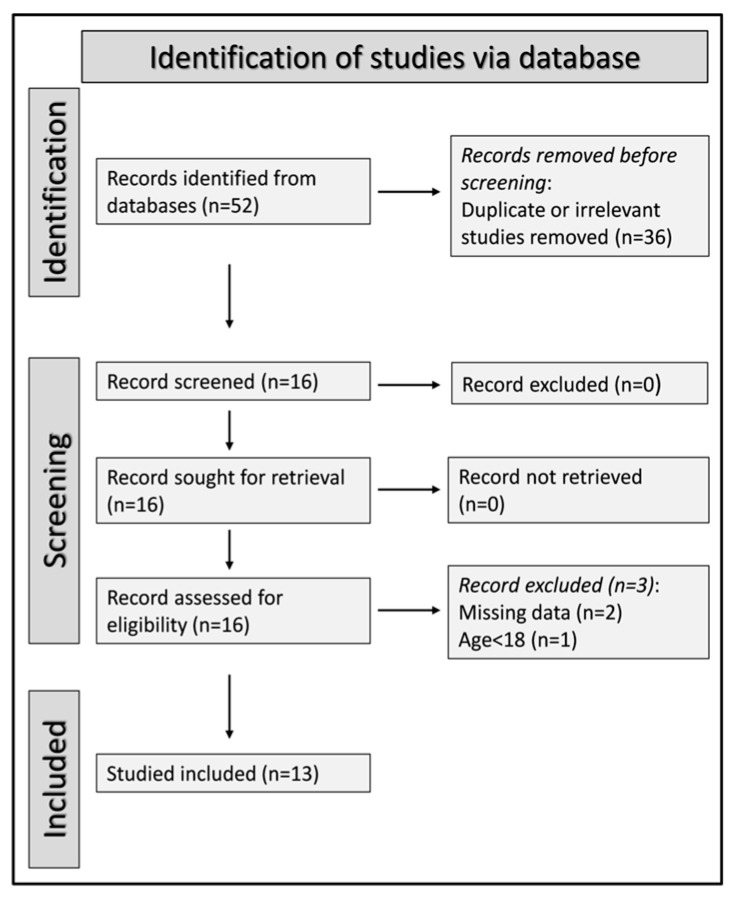
PRISMA 2020 flow chart of study selection.

After initially identifying 52 articles, 36 were removed (either duplicates or irrelevant). After full-text review, a further three were excluded (missing data: two studies; participants aged <18 years: one study), leaving 13 articles, all retrospective studies, for final analysis (Figure 1) [25,40,41,42,43,44,45,46,47,48,49,50,51]. Clinical endpoints included mortality (11 study groups) [25,40,43,44,47,48,49,50,51], and the following measures of disease severity: transfer to the intensive care unit (two study groups) [42,46], invasive mechanical ventilation (two study groups) [44,45], prolonged hospital stay (one study group) [41], acute limb ischemia (one study group) [42], deep vein thrombosis (one study group) [47], and acute pulmonary embolism (one study group) [47]. The AISI was measured on admission in all studies. The risk of bias and the initial certainty of evidence (case-control design; rating 2, ⊕⊕⊖⊖) were low in all studies (Supplementary Table S3) [25,40,41,42,43,44,45,46,47,48,49,50,51].

### 3.2. Standardized Mean Differences

Eleven studies (14 groups) reported the AISI in 1600 non-severe disease/survivor (NSDS, mean age 68 years, 57% males) and 4521 severe disease/non-survivor patients (SDNS, mean age 62 years, 56% males) [25,40,41,42,43,44,46,47,48,50,51]. Five studies were conducted in Europe [25,40,41,42,47], four in Asia [43,48,50,51], one in America [44], and one in Africa [46] (Table 1).

**Figure 2:**
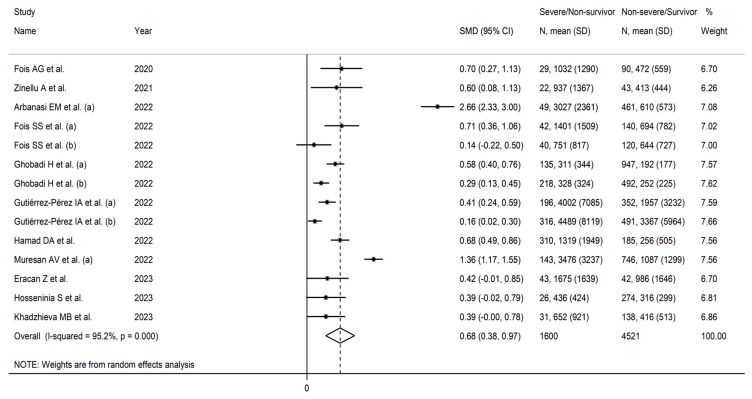
Forest plot of AISI values in NSDS and SDNS patients [25,40,41,42,43,44,46,47,48,50,51].

**Figure 3:**
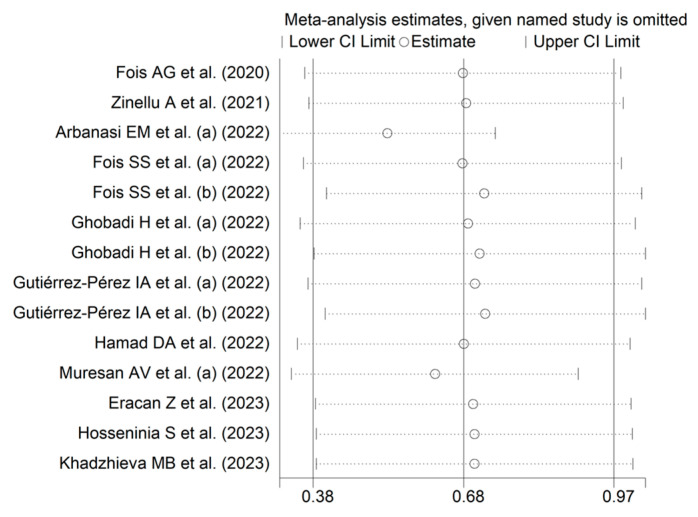
Sensitivity analysis of studies reporting the AISI in COVID-19 [25,40,41,42,43,44,46,47,48,50,51].

Random effects models were used because of the high heterogeneity observed (I^2^ = 95.2%, *p* < 0.001). SDNS patients had significantly higher AISI values vs. NSDS (SMD = 0.68, 95% CI 0.38 to 0.97, *p* < 0.001; Figure 2). The pooled SMD values were stable in sensitivity analysis (range 0.53–0.72; Figure 3).

**Figure 4:**
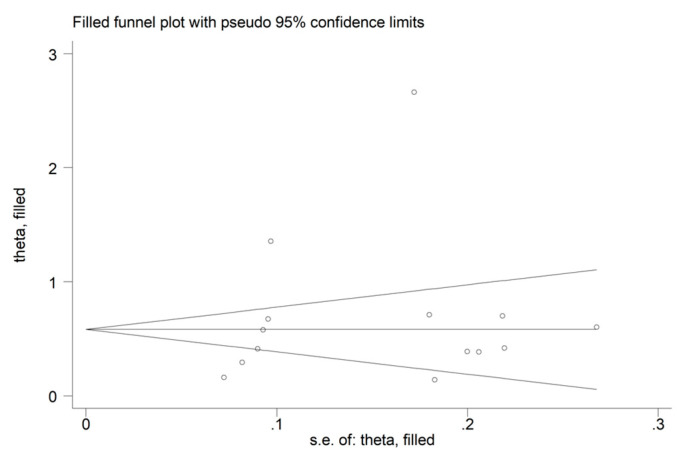
Funnel plot of studies investigating AISI values in COVID-19 patients after “trimming-and-filling”.

No publication bias was detected with either the Begg’s test (*p* = 0.23), the Egger’s test (*p* = 0.46), or the “trim-and-fill” method (Figure 4). However, the funnel plot revealed the distortive effect of one study [42]. Its removal did not tangibly influence the effect size (SMD = 0.53, 95% CI 0.32 to 0.74, *p* < 0.001; I^2^ = 89.9%, *p* < 0.001).

**Figure 5:**
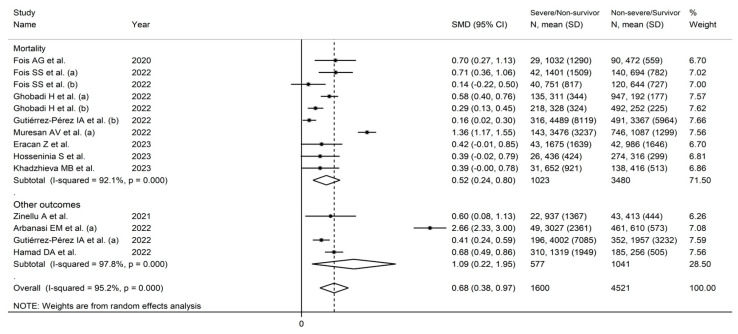
Forest plot of studies of AISI in COVID-19 according to clinical endpoint [25,40,41,42,43,44,46,47,48,50,51].

**Figure 6:**
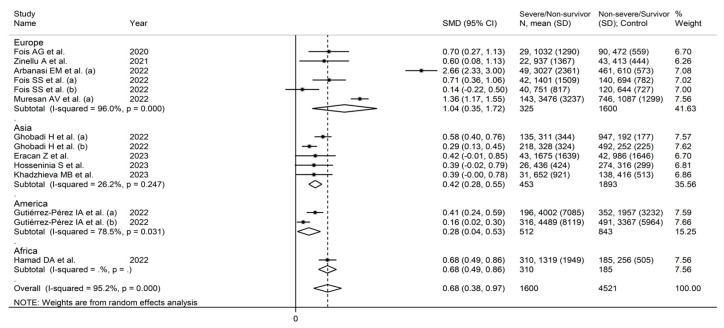
Forest plot of studies examining AISI in COVID-19 according to study continent [25,40,41,42,43,44,46,47,48,50,51].

In meta-regression, there were non-significant correlations between the effect size and age (t = 0.25, *p* = 0.81), proportion of males (t = 0.63, *p* = 0.55), publication year (t = −0.76, *p* = 0.46), and sample size (t = 0.70, *p* = 0.50). In sub-group analysis, there were non-significant differences (*p* = 0.15) in SMD between studies reporting mortality (SMD = 0.52, 95% CI 0.24 to 0.80, *p* < 0.001; I^2^ = 92.1%, *p* < 0.001) and those reporting measures of disease severity (SMD = 1.09, 95% CI 0.22 to 1.95, *p* = 0.014; I^2^ = 97.8%, *p* < 0.001; Figure 5). Similarly, non-significant differences (*p* = 0.19) were observed in the pooled SMD between European (SMD = 1.04, 95% CI 0.35 to 1.72, *p* = 0.003; I^2^ = 96.0%, *p* < 0.001), Asian (SMD = 0.42, 95% CI 0.28 to 0.55, *p* < 0.001; I^2^ = 26.2%, *p* < 0.001), and American studies (SMD = 0.28, 95% CI 0.04 to 0.53, *p* = 0.024; I^2^ = 78.5%, *p* < 0.001; Figure 6). However, the variance was substantially reduced in the Asian subgroup (I^2^ = 26.5%).

The level of certainty remained low (rating 2, ⊕⊕⊖⊖) after considering the low risk of bias in all studies, the high but partly explainable heterogeneity, the lack of indirectness, the relatively low imprecision, the moderate effect size, and the absence of publication bias.

### 3.3. Odds Ratios

Five studies (including nine groups) reported multivariate logistic associations between the AISI and disease severity/survival as ORs in 5794 COVID-19 patients (59% males, mean age 66 years) [42,44,45,46,47]. Endpoints included mortality (two studies) [44,47], acute limb ischemia [42], invasive mechanical ventilation (two studies) [44,45], deep vein thrombosis (one study) [47], and acute pulmonary embolism (one study) [47]. Three studies were conducted in Europe [42,45,47], one in America [44], and one in Africa (Table 2) [46].

**Figure 7:**
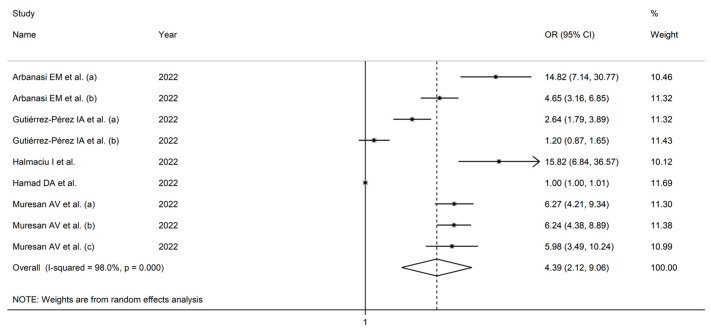
Forest plot of studies examining the association between AISI and disease severity/survival in COVID-19 with odds ratio [42,44,45,46,47].

**Figure 8:**
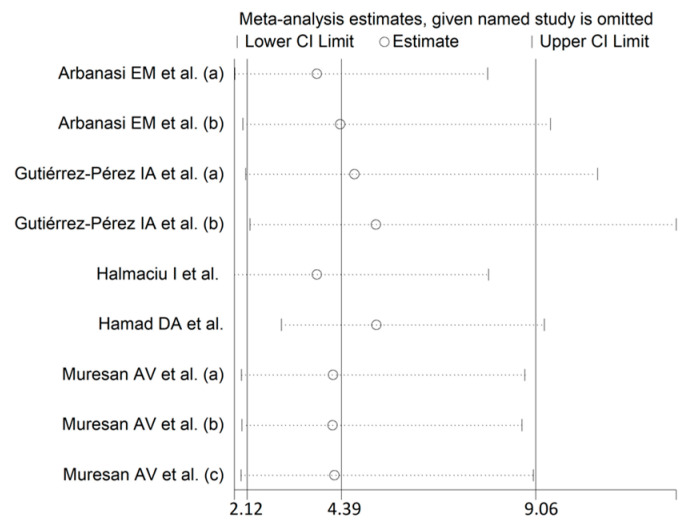
Sensitivity analysis of the association between the AISI and COVID-19 using odds ratios [42,44,45,46,47].

Using random-effects models (I^2^ = 98.0%, *p* < 0.001), higher AISI values were significantly associated with SDNS (OR = 4.39, 95% CI 2.12 to 9.06, *p* < 0.001; Figure 7). The effect size was stable in sensitivity analysis (range 3.79–5.23; Figure 8).

**Figure 9:**
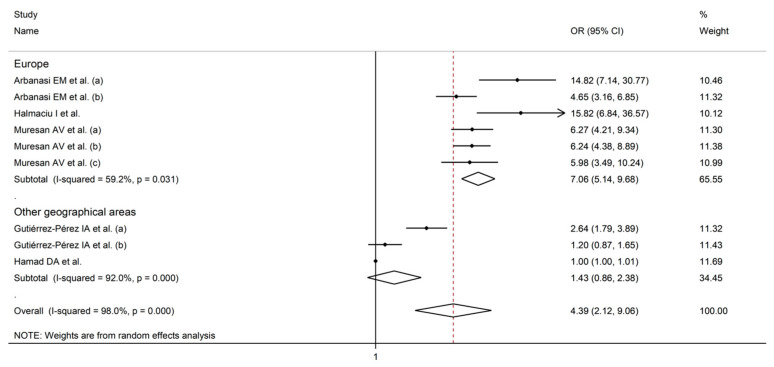
Forest plot of studies examining the odds ratio of AISI and COVID-19 severity/survival according to study continent [42,44,45,46,47].

Assessment of publication bias and meta-regression analysis were prevented by the relatively small number of studies. In sub-group analysis, the effect size was significantly different in studies conducted in Europe (OR = 7.06, 95 % CI 5.14 to 9.68, *p* < 0.001; I^2^ = 59.2%, *p* = 0.03), but not in other geographical areas (OR = 1.43, 95 % CI 0.86 to 2.38, *p* = 0.17; I^2^ = 92.0%, *p* < 0.001; Figure 9), with a lower between-study variance in the former subgroup.

The level of certainty remained low (rating 2, ⊕⊕⊖⊖) after considering the low risk of bias in all studies, the high but partly explainable heterogeneity, the lack of indirectness, the relatively low imprecision, the relatively large effect size (OR = 4.39; upgrade one level), and the lack of assessment of publication bias (downgrade one level).

### 3.4. Hazard Ratios

Three studies reported multivariate logistic associations between the AISI and mortality as HRs in 504 COVID-19 patients (57% males, mean age 70 years) [40,48,50]. Two studies were conducted in Asia [48,50], and one in Europe (Table 2) [40]. The risk of bias was low in all studies (Supplementary Table S3) [40,48,50].

**Figure 10:**
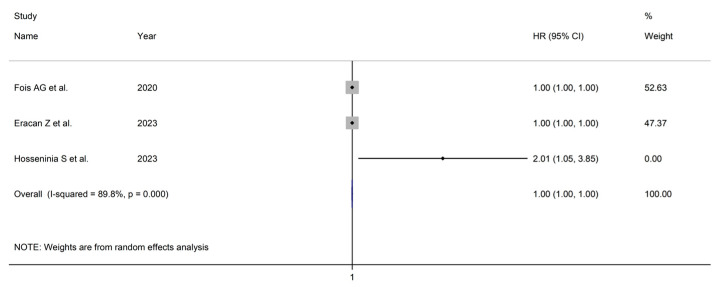
Forest plot of studies examining the association between AISI and COVID-19 severity/survival using hazard ratio [40,48,50].

Using random-effects models (I^2^ = 89.8%, *p* < 0.001), the AISI was not associated with mortality (HR = 1.000, 95% CI 0.999 to 1.002, *p* = 0.39; Figure 10). Assessment of publication bias, meta-regression, and subgroup analyses was not possible because of the relatively small number of studies.

The level of certainty was downgraded to extremely low (rating 0, ⊖⊖⊖⊖) after considering the high heterogeneity, the relatively high imprecision, and the lack of assessment of publication bias.

### 3.5. Diagnostic Accuracy for Prediction of Severe Disease or Death

Eleven studies (including 15 patient groups) reported the diagnostic accuracy of the AISI towards severe disease or death in 7427 COVID-19 patients (56% males, mean age 58 years) (Table 3) [25,40,41,42,43,45,46,47,48,49,50]. Six studies were conducted in Europe [25,40,41,42,45,47], four in Asia [43,48,49,50], and one in Africa [46]. Endpoints included mortality (eight study groups) [25,40,43,47,48,49,50], admission to the intensive care unit (two study groups) [42,46], prolonged hospital stay (one study group) [41], acute limb ischemia (one study group) [42], invasive mechanical ventilation (one study group) [45], deep vein thrombosis (one study group) [47], and acute pulmonary embolism (one study group) [47]. The risk of bias was low in all studies (Supplementary Table S3) [25,40,41,42,43,45,46,47,48,49,50].

**Figure 11:**
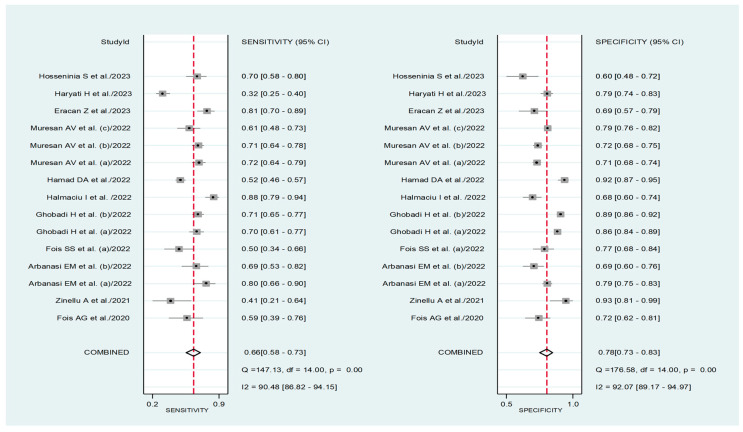
Forest plot for the pooled estimates of sensitivity and specificity of AISI values for adverse outcome prediction [25,40,41,42,43,45,46,47,48,49,50].

**Figure 12:**
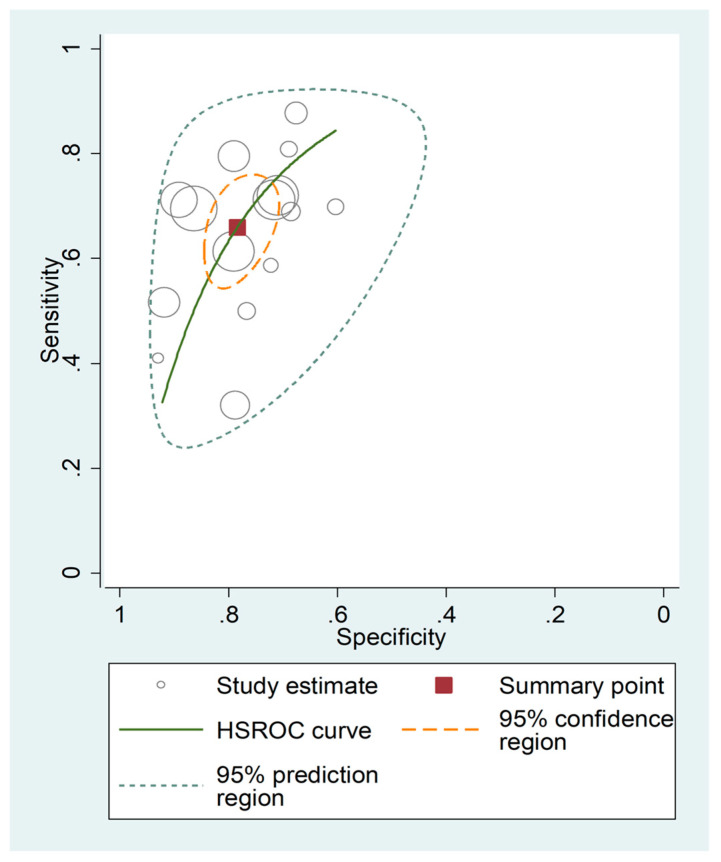
SROC curve of AISI values for adverse outcome prediction.

**Figure 13:**
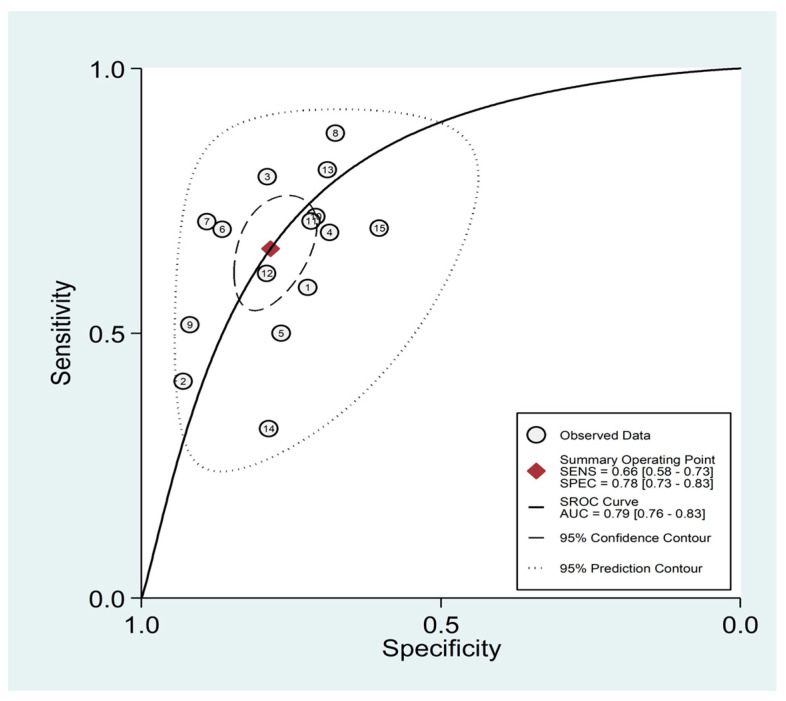
Empirical Bayes estimates of HSROC curve for adverse outcome prediction.

**Figure 14:**
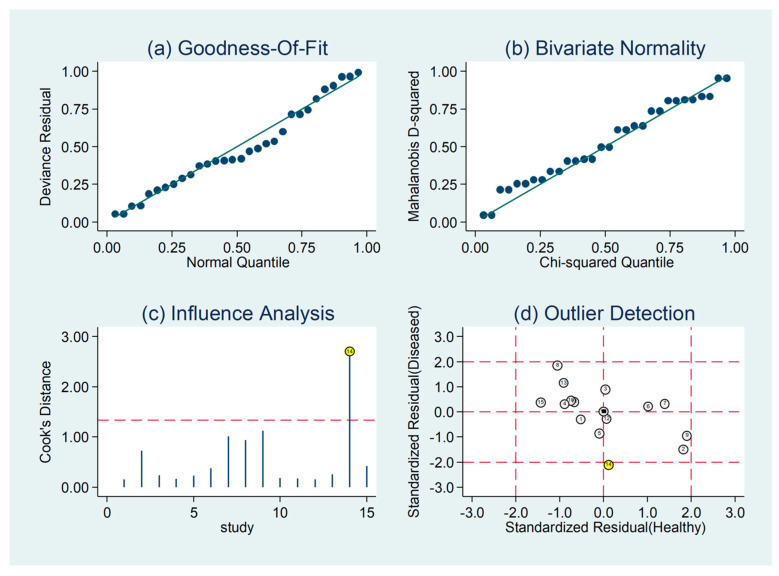
Residual-based goodness-of-fit, bivariate normality, influence, and outlier detection.

The pooled sensitivity and specificity of the AISI for severe disease/death were 0.66 (95% CI 0.58 to 0.73) and 0.78 (95% CI 0.73 to 0.83), respectively (Figure 11). The AUC was 0.79 (95% CI 0.76 to 0.83), with sensitivity of 0.66 and specificity of 0.78 (Figure 12). The empirical Bayes estimates in HSROC analysis are shown in Figure 13. The midas command was used to evaluate the quantile plot of residual-based goodness-of-fit, the Chi-squared probability plot of squared Mahalanobis distances for assessment of the bivariate normality assumption, the spikeplot for checking for particularly influential observations using Cook’s distance, and a scatter plot for checking for outliers using standardized predicted random effects (Figure 14). The analysis identified one outlier [49]. Its removal resulted in an AUC value of 0.80, a sensitivity of 0.69, and a specificity of 0.79.

**Figure 15:**
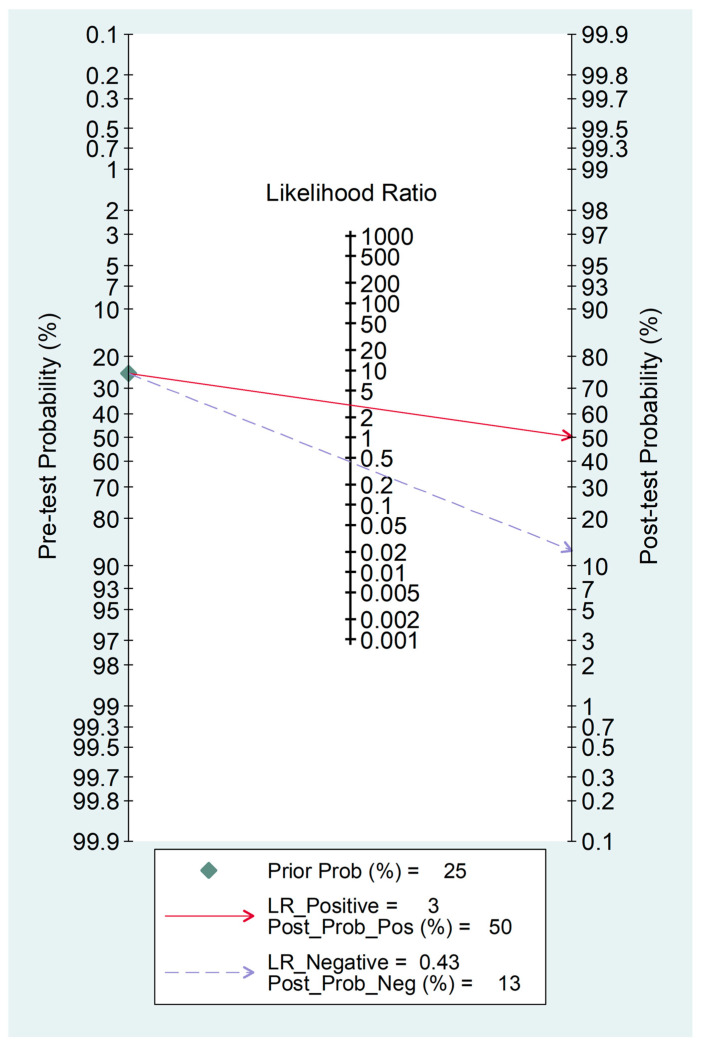
Fagan’s nomogram of AISI for adverse outcome prediction.

The Fagan’s nomogram, generated to assess the potential clinical utility of the AISI, showed that, assuming a pre-test probability of 25% for adverse outcomes, the post-test probability was 50% with relatively high AISI values and 13% with relatively low AISI values (Figure 15).

**Figure 16:**
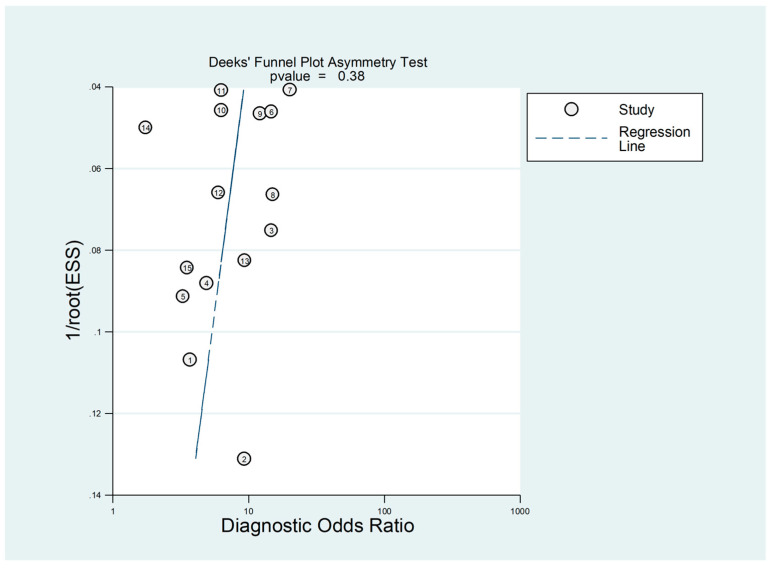
Deeks funnel plot asymmetry test.

There was no significant publication bias (*p* = 0.38) according to the Deeks funnel plot asymmetry test (Figure 16).

**Figure 17:**
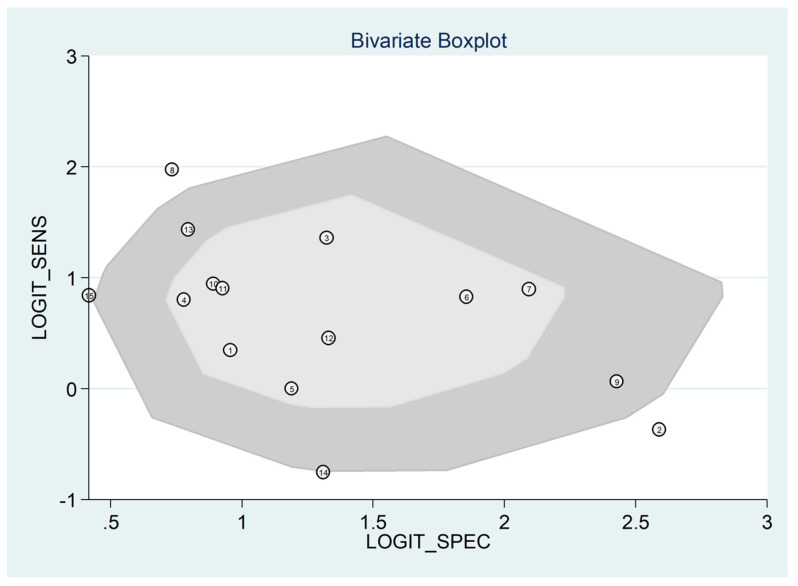
Bivariate boxplot exploring heterogeneity.

**Figure 18:**
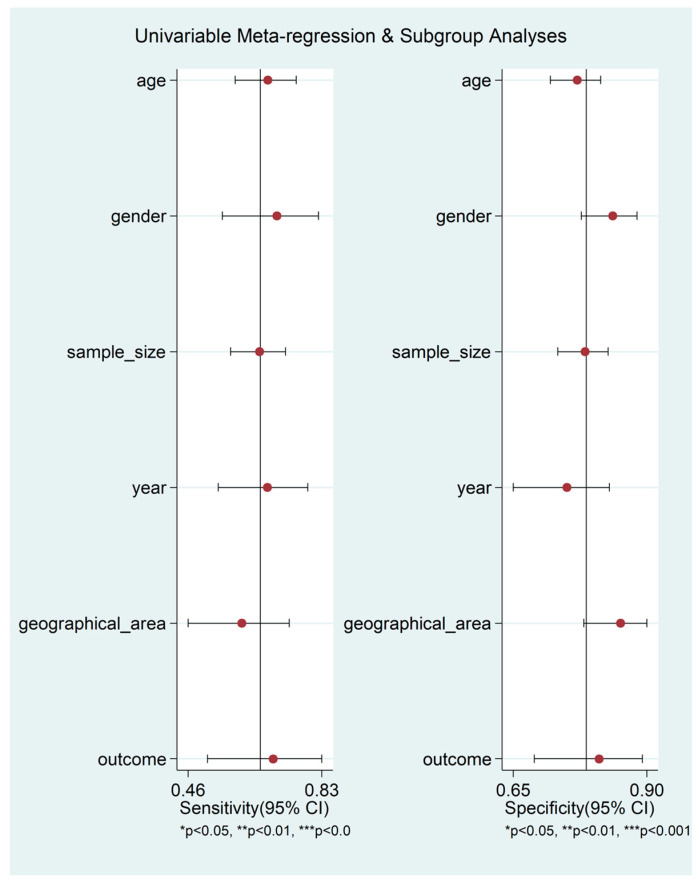
Forest plots of sensitivity and specificity for the study level covariates included in the univariate meta-regression model.

The HSROC curve (Figure 12) was symmetric, given the negative correlation coefficient between logit transformed sensitivity and specificity (HSROC model; −0.634, 95% CI −0.897 to −0.432), and the non-significant (*p* = 0.50) symmetry parameter β (0.209, 95% CI −0.394 to 0.812; data not reported in tables or graphs). This suggests the absence of between-study heterogeneity [34,36]. However, the visual representation of SROC (Figure 12) suggests moderate heterogeneity (95% CI 0.76 to 0.83). Using midas, the pooled sensitivity and specificity showed an inconsistency (I^2^) of 90.48 and 92.07%, respectively (Figure 11). Using the bivariate boxplot with logit_Se and logit_Sp (Figure 17), four studies fell outside the circles [41,45,49,50], which indicates the presence of heterogeneity across studies. None of the investigated parameters exhibited significant associations with the effect size for sensitivity or specificity (Figure 18).

## 4. Discussion

In our study, the AISI values on admission were significantly higher in hospitalized SDNS vs. NSDS COVID-19 patients. The AISI between-group differences were significant when expressed either as SMDs or as ORs, whereas the lack of significant differences in studies reporting HRs is likely to be secondary to their limited number (*n* = 3). Importantly, the AISI exhibited good diagnostic performance towards severe disease or mortality, with an AUC of 0.79. Sensitivity analysis showed that the results of the meta-analysis were stable. There were non-significant associations in meta-regression between the effect size and either age, sex, publication year, or sample size. In particular, the absence of significant associations with the year of publication suggests that the capacity of the AISI to discriminate between COVID-19 patients with different disease severity and survival status persisted during different phases of the COVID-19 pandemic, regardless of study participants with different vaccines, viral variants, and treatments. In subgroup analysis, there were differences in effect size with study continent, particularly for ORs, indicating possible ethnicity-related effects on the association between the AISI and COVID-19.

Compared to other indexes of inflammation derived from haematological parameters, e.g., NLR and the platelet-to-lymphocyte ratio (PLR), the AISI is calculated using information from four types of blood cell that are involved in inflammation, i.e., neutrophils, monocytes, platelets, and lymphocytes. The AISI was initially investigated in 2018 to predict outcomes in surgical patients [19]. Since then, studies have assessed the potential clinical utility of the AISI in patients with other disease states characterized by a systemic pro-inflammatory state, such as macular degeneration [20], idiopathic pulmonary fibrosis [21,23], and cancer [24]. Notably, in patients with idiopathic pulmonary fibrosis, the capacity of the AISI to predict for four-year survival was superior to white blood cell, neutrophil, monocyte, lymphocyte, and platelet count taken singly, and the NLR and the PLR, further supporting its potential clinical utility [23]. Over the last three years, an increasing number of studies have also investigated the AISI in patients with COVID-19, given the critical pathophysiological and prognostic role of excess systemic inflammation in terms of severe clinical manifestations, multiorgan involvement, requirement for intensive care, prolonged hospitalization, and in-hospital mortality [1,2,3,4,5,6,7,8,9,10].

The comprehensive appraisal of the available evidence strongly supports the concept that assessing the AISI at the time of admission can provide useful information to rapidly direct subgroups of patients with COVID-19 to different treatment and monitoring pathways. This proposition is further supported by the observed pooled AUC value and the Fagan’s nomogram, which indicated a wide separation of the post-test probability of severe disease/death compared to the pre-test probability, according to whether the admission AISI values were relatively low or relatively high [52]. However, it should be emphasised that further studies are required before routinely using the AISI to assess COVID-19 patients. Specifically, prospective studies should investigate whether the AISI can predict, with or without other clinical parameters, ethnicity, and specific comorbidities, disease severity and mortality in this group, in a similar way to other conditions [53].

One limitation of our study is the moderate-to-high between-study heterogeneity. However, we identified specific sources of heterogeneity when investigating the SMD (study continent) and the OR (study continent). Furthermore, publication bias could not be assessed with the OR and the HR because of the limited number of relevant studies. In contrast, significant strengths are the comprehensive assessment of the significance of the AISI with meta-regression and subgroup analysis, SROC, and Fagan’s nomogram.

## 5. Conclusions

In our study, higher AISI values on admission were significantly associated with severe disease/death in COVID-19. Further studies in patients with a wide range of comorbidities and ethnic backgrounds are warranted to determine whether the AISI can routinely assist with discriminating between COVID-19 patients with different severity and outcomes in order to optimize management and outcomes.

## Figures and Tables

**Table 1:** Studies reporting AISI in COVID-19 patients with NSDS and SDNS.

	Non-Severe Disease or Survivor (NSDS)				Severe Disease or Non-Survivor (SDNS)				Outcome
Study	*n*	Age (Years)	M/F	AISI (Mean ± SD)	*n*	Age (Years)	M/F	AISI (Mean ± SD)	
Fois AG et al., 2020, Italy [40]	90	68	56/34	472 ± 559	29	80	21/8	1032 ± 1290	Mortality
Zinellu A et al., 2021, Italy [41]	43	66	27/16	413 ± 444	22	69	16/6	937 ± 1367	Length of stay
Arbanasi EM et al. (a) 2022, Romania [42]	461	70	284/177	610 ± 573	49	74	21/28	3027 ± 2361	ALI
Fois SS et al. (a), 2022, Italy [25]	140	68	91/49	694 ± 782	42	82	32/10	1401 ± 1509	Mortality
Fois SS et al. (b), 2022, Italy [25]	120	73	66/54	644 ± 727	40	82	20/20	751 ± 817	Mortality
Ghobadi H et al. (a) 2022, Iran [43]	947	48	548/399	192 ± 177	135	54	88/47	311 ± 344	Mortality
Ghobadi H et al. (b) 2022, Iran [43]	492	76	238/254	252 ± 225	218	78	114/104	328 ± 324	Mortality
Gutiérrez-Pérez IA et al. (a) 2022, Mexico [44]	352	NR	NR	1957 ± 3232	196	NR	NR	4002 ± 7085	IMV
Gutiérrez-Pérez IA et al. (b) 2022, Mexico [44]	491	NR	NR	3367 ± 5964	316	NR	NR	4489 ± 8119	Mortality
Hamad DA et al., 2022, Egypt [46]	185	33	91/94	256 ± 505	310	58	181/129	1319 ± 1949	ICU admission
Muresan AV et al. (a), 2022 Romania [47]	746	70	397/349	1087 ± 1299	143	72	77/66	3476 ± 3237	Mortality
Ercan Z et al., 2023, Turkey [48]	42	74	23/19	986 ± 1646	43	73	16/27	1675 ± 1639	Mortality
Hosseninia S et al., 2023, Romania [50]	274	68	70/53	316 ± 299	26	73	28/18	436 ± 424	Mortality
Khadzhieva MB et al., 2023, Russia [51]	138	57	73/65	416 ± 513	31	62	18/13	652 ± 921	Mortality

Legend: AISI, aggregate index of systemic inflammation; ALI, acute limb ischemia; F, female; ICU, intensive care unit; IMV, invasive mechanical ventilation; M, male; NR, not reported.

**Table 2:** Studies reporting associations between AISI and disease severity/survival in COVID-19 with odds ratio or hazard ratio.

Study	Design	*n*	Age (Years)	M/F	OR	95% CI	Outcome
Arbanasi EM et al. (a) 2022, Romania [42]	R	510	70	305/205	14.82	7.14–30.77	Acute limb ischemia
Arbanasi EM et al. (b) 2022, Romania [42]	R	510	70	305/205	4.65	3.16–6.85	Admission to intensive care unit
Gutiérrez-Pérez IA et al. (a) 2022, Mexico [44]	R	548	NR	352/196	2.64	1.79–3.89	Invasive mechanical ventilation
Gutiérrez-Pérez IA et al. (b) 2022, Mexico [44]	R	807	NR	491/316	1.2	0.87–1.65	Mortality
Halmaciu I et al., 2022, Romania [45]	R	267	71	159/108	15.82	6.86–36.65	Invasive mechanical ventilation
Hamad DA et al., 2022, Egypt [46]	R	485	49	272/213	1.003	1.001–1.005	Admission to intensive care unit
Muresan AV et al. (a) 2022, Romania [47]	R	889	71	474/415	6.27	4.21–9.34	Mortality
Muresan AV et al. (b) 2022, Romania [47]	R	889	71	474/415	6.24	4.38–8.89	Deep vein thrombosis
Muresan AV et al. (c) 2022, Romania [47]	R	889	71	474/415	5.98	3.49–10.24	Acute pulmonary embolism
Study	Design	*n*	Age (Years)	M/F	HR	95% CI	Outcome
Fois AG et al., 2020, Italy [40]	R	119	72	77/42	1	1–1.0001	Mortality
Ercan Z et al., 2023, Turkey [48]	R	85	72	39/46	1.001	1–1.001	Mortality
Hosseninia S et al., 2023, Iran [50]	R	300	69	98/71	2.01	1.048–3.855	Mortality

Legend: CI, confidence interval; F, female; HR, hazard ratio; M, male; NR, not reported; OR, odds ratio; retrospective.

**Table 3:** Studies reporting the diagnostic accuracy of AISI for predicting severe disease/death in COVID-19.

Study	Design	*n*	AUC (95% CI)	Cut-Off	Sensitivity (%)	Specificity (%)	Outcome
Fois AG et al., 2020, Italy [40]	R	119	0.640	798	0.59	0.72	Mortality
			0.546–0.726				
Zinellu A et al., 2021, Italy [41]	R	65	0.674	1153	0.4	0.93	Length of stay
			0.547–0.786				
Arbanasi EM et al. (a) 2022, Romania [42]	R	510	0.851	1296.62	0.8	0.79	Acute limb ischemia
			0.789–0.913				
Arbanasi EM et al. (b) 2022, Romania [42]	R	510	0.738	650.58	0.679	0.687	Admission to intensive care unit
			0.692–0.783				
Fois SS et al. (a) 2022, Italy [25]	R	182	0.645	1282	0.51	0.77	Mortality
			0.570–0.715				
Ghobadi H et al. (a) 2022, Iran [43]	R	1082	0.871	492	0.693	0.865	Mortality
			0.849–0.890				
Ghobadi H et al. (b) 2022, Iran [43]	R	710	0.826	518	0.711	0.89	Mortality
			0.796–0.853				
Halmaciu I et al., 2022, Romania [45]	R	267	0.813	994.76	0.883	0.676	Invasive mechanical ventilation
			0.754–0.871				
Hamad DA et al., 2022, Egypt [46]	R	485	0.807	729	0.517	0.919	Admission to intensive care unit
			0.767–0.846				
Muresan AV et al. (a) 2022, Romania [47]	R	889	0.780	1696.18	0.72	0.709	Mortality
			0.740–0.821				
Muresan AV et al. (b) 2022, Romania [47]	R	889	0.784	1605.4	0.712	0.716	Deep vein thrombosis
			0.745–0.823				
Muresan AV et al. (c) 2022, Romania [47]	R	889	0.750	2769.85	0.613	0.791	Acute pulmonary oedema
			0.684–0.816				
Ercan Z et al., 2023, Turkey [48]	R	85	0.820	621.1	0.81	0.691	Mortality
			0.733–0.903				
Haryati H et al., 2023, Indonesia [49]	R	445	0.558	1422	0.32	0.788	Mortality
			0.510–0.614				
Hosseninia S et al., 2023, Iran [50]	R	300	0.630	260	0.696	0.61	Mortality
			0.552–0.703				

Legend: AUC, area under the curve; R, retrospective.

## Data Availability

The relevant data are available from A.Z. upon reasonable request.

## Supplementary Materials

The following supporting information can be downloaded at: https://www.mdpi.com/article/10.3390/jcm12144584/s1, Table S1: PRISMA 2020 for abstracts checklist; Table S2: PRISMA 2020 checklist; Table S3: The Joanna Briggs Institute Critical Appraisal Checklist. References [25,40,41,42,43,44,45,46,47,48,49,50,51] are cited in the Supplementary Materials.
