# Supplementary material for: Aggregate Index of Systemic Inflammation (AISI), Disease Severity, and Mortality in COVID-19: A Systematic Review and Meta-Analysis
Source: J Clin Med. 2023 Jul 10;12(14):4584. doi: 10.3390/jcm12144584 (PMC10381001; doi:10.3390/jcm12144584)
Supplement: Supplementary file 1 [file jcm-12-04584-s001.zip › Supplementary_Table_S3.pdf]

Supplementary Table S3. The Joanna Briggs Institute critical appraisal checklist.

Study	Were the groups comparable other than the presence of disease in cases or the absence of disease in controls?	Were the same criteria used for identification of cases and controls?	Was the exposure measured in a valid and reliable way?	Was exposure measured in the same way for cases and controls?	Were confounding factors identified?	Were strategies to deal with confounding factors stated?	Were the outcomes measured in a valid and reliable way?	Was the exposure period of interest long enough to be meaningful?	Was appropriate statistical analysis used?	Risk of bias
Fois AG [1]	No	Yes	Yes	Yes	Yes	Yes	Yes	Yes	Yes	Low
Zinellu A [2]	Yes	Yes	Yes	Yes	Yes	Yes	Yes	Yes	Yes	Low
Arbanasi EM [3]	No	Yes	Yes	Yes	Yes	Yes	Yes	Yes	Yes	Low
Fois SS [4]	No	Yes	Yes	Yes	Yes	Yes	Yes	Yes	Yes	Low
Ghobadi H [5]	No	Yes	Yes	Yes	Yes	Yes	Yes	Yes	Yes	Low
Gutiérrez-Pérez IA [6]	No	Yes	Yes	Yes	Yes	Yes	Yes	Yes	Yes	Low
Halmaciu I [7]	No	Yes	Yes	Yes	Yes	Yes	Yes	Yes	Yes	Low
Hamad DA [8]	No	Yes	Yes	Yes	Yes	Yes	Yes	Yes	Yes	Low
Muresan AV [9]	No	Yes	Yes	Yes	Yes	Yes	Yes	Yes	Yes	Low
Ercan Z et [10]	Yes	Yes	Yes	Yes	No	No	Yes	Yes	Yes	Low
Haryati [11]	Yes	Yes	Yes	Yes	Yes	Yes	Yes	Yes	Yes	Low
Hosseninia S [12]	No	Yes	Yes	Yes	Yes	Yes	Yes	Yes	Yes	Low
Khadzhieva MB [13]	No	Yes	Yes	Yes	Yes	Yes	Yes	Yes	Yes	Low

References

1. Fois, A.G.; Paliogiannis, P.; Scano, V.; Cau, S.; Babudieri, S.; Perra, R.; Ruzzittu, G.; Zinellu, E.; Pirina, P.; Carru, C.; et al. The Systemic Inflammation Index on Admission Predicts In-Hospital Mortality in COVID-19 Patients. *Molecules* **2020**, *25*, doi:10.3390/molecules25235725.
2. Zinellu, A.; Scano, V.; Masotto, E.; De Riu, G.; Vaira, L.A.; Carru, C.; Pirina, P.; Babudieri, S.; Mangoni, A.A.; Fois, A.G. The Systemic Inflammation Index on admission is independently associated with length of stay in hospitalized COVID-19 patients. *Minerva Respiratory Medicine* **2021**, *60*, doi:10.23736/s2784-8477.21.01932-5.
3. Arbanasi, E.M.; Halmaciu, I.; Kaller, R.; Muresan, A.V.; Arbanasi, E.M.; Suciu, B.A.; Cosarca, C.M.; Cojocaru, II; Melinte, R.M.; Russu, E. Systemic Inflammatory Biomarkers and Chest CT Findings as Predictors of Acute Limb Ischemia Risk, Intensive Care Unit Admission, and Mortality in COVID-19 Patients. *Diagnostics (Basel)* **2022**, *12*, doi:10.3390/diagnostics12102379.
4. Fois, S.S.; Zinellu, E.; Zinellu, A.; Merella, M.; Pau, M.C.; Carru, C.; Fois, A.G.; Pirina, P. Comparison of Clinical Features, Complete Blood Count Parameters, and Outcomes between Two Distinct Waves of COVID-19: A Monocentric Report from Italy. *Healthcare (Basel)* **2022**, *10*, doi:10.3390/healthcare10122427.
5. Ghobadi, H.; Mohammadshahi, J.; Javaheri, N.; Fouladi, N.; Mirzazadeh, Y.; Aslani, M.R. Role of leukocytes and systemic inflammation indexes (NLR, PLR, MLP, dNLR, NLPR, AISI, SIR-I, and SII) on admission predicts in-hospital mortality in non-elderly and elderly COVID-19 patients. *Front Med (Lausanne)* **2022**, *9*, 916453, doi:10.3389/fmed.2022.916453.
6. Gutierrez-Perez, I.A.; Buendia-Roldan, I.; Perez-Rubio, G.; Chavez-Galan, L.; Hernandez-Zenteno, R.J.; Aguilar-Duran, H.; Fricke-Galindo, I.; Zaragoza-Garcia, O.; Falfan-Valencia, R.; Guzman-Guzman, I.P. Outcome predictors in COVID-19: An analysis of emergent systemic inflammation indices in Mexican population. *Front Med (Lausanne)* **2022**, *9*, 1000147, doi:10.3389/fmed.2022.1000147.
7. Halmaciu, I.; Arbanasi, E.M.; Kaller, R.; Muresan, A.V.; Arbanasi, E.M.; Bacalbasa, N.; Suciu, B.A.; Cojocaru, II; Runcan, A.I.; Grosu, F.; et al. Chest CT Severity Score and Systemic Inflammatory Biomarkers as Predictors of the Need for Invasive Mechanical Ventilation and of COVID-19 Patients' Mortality. *Diagnostics (Basel)* **2022**, *12*, doi:10.3390/diagnostics12092089.
8. Hamad, D.A.; Aly, M.M.; Abdelhameid, M.A.; Ahmed, S.A.; Shaltout, A.S.; Abdel-Moniem, A.E.; Ragheb, A.M.R.; Attia, M.N.; Meshref, T.S. Combined Blood Indexes of Systemic Inflammation as a Mirror to Admission to Intensive Care Unit in COVID-19 Patients: A Multicentric Study. *J Epidemiol Glob Health* **2022**, *12*, 64-73, doi:10.1007/s44197-021-00021-5.
9. Muresan, A.V.; Halmaciu, I.; Arbanasi, E.M.; Kaller, R.; Arbanasi, E.M.; Budisca, O.A.; Melinte, R.M.; Vunvulea, V.; Filep, R.C.; Marginean, L.; et al. Prognostic Nutritional Index, Controlling Nutritional Status (CONUT) Score, and Inflammatory Biomarkers as Predictors of Deep Vein Thrombosis, Acute Pulmonary Embolism, and

Mortality in COVID-19 Patients. *Diagnostics (Basel)* **2022**, *12*, doi:10.3390/diagnostics12112757.

10. Ercan, Z.; Evren Oztop, K.; Pinar, M.; Varim, C.; Dheir, H.; Karacaer, C.; Yaylaci, S.; Bilal Genc, A.; Cekic, D.; Nalbant, A.; et al. The aggregate index of systemic inflammation may predict mortality in COVID-19 patients with chronic renal failure. *Eur Rev Med Pharmacol Sci* **2023**, *27*, 3747-3752, doi:10.26355/eurrev_202304_32173.

11. Haryati, H.; Wicaksono, B.; Syahadatina, M. Complete blood count derived inflammation indexes predict outcome in COVID-19 patients: a study in Indonesia. *J Infect Dev Ctries* **2023**, *17*, 319-326, doi:10.3855/jidc.16527.

12. Hosseninia, S.; Ghobadi, H.; Garjani, K.; Hosseini, S.A.H.; Aslani, M.R. Aggregate index of systemic inflammation (AISI) in admission as a reliable predictor of mortality in COPD patients with COVID-19. *BMC Pulm Med* **2023**, *23*, 107, doi:10.1186/s12890-023-02397-5.

13. Khadzhieva, M.B.; Gracheva, A.S.; Belopolskaya, O.B.; Chursinova, Y.V.; Redkin, I.V.; Pisarev, M.V.; Kuzovlev, A.N. Serial Changes in Blood-Cell-Count-Derived and CRP-Derived Inflammatory Indices of COVID-19 Patients. *Diagnostics (Basel)* **2023**, *13*, doi:10.3390/diagnostics13040746.
